# 3D Swin Transformer for patient-specific proton dose prediction of brain cancer patients

**DOI:** 10.2340/1651-226X.2025.43969

**Published:** 2025-11-02

**Authors:** Anne Haahr Andresen, Slávka Lukacova, Yasmin Lassen-Ramshad, Christian Rønn Hansen, Jesper Folsted Kallehauge

**Affiliations:** a Department of Clinical Medicine, Arhus University, Aarhus, Denmark; b Danish Centre for Particle Therapy, Aarhus University Hospital, Aarhus, Denmark; c Department of Oncology, Aarhus University Hospital, Aarhus, Denmark; d Department of Oncology, Odense University Hospital, Odense, Denmark; e Institute of Clinical Research, University of Southern Denmark, Odense, Denmark

**Keywords:** Artificial intelligence, deep learning, proton therapy, brain cancer, dose prediction

## Abstract

**Background and purpose:**

Accurate dose plans in proton radiotherapy with consistent target in complex anatomical regions such as the brain are crucial. This study investigates a Swin Transformer-based deep learning model for voxel-wise dose prediction in brain cancer proton therapy, evaluating its spatial and dosimetric fidelity against clinically delivered plans.

**Patient/material and methods:**

A cohort of 206 patients with primary brain tumors were retrospectively analyzed. Dual-energy computed tomography (CT) scans, clinical contours, and corresponding proton dose plans were used to train and test a 3D Swin Transformer integrated within a UNet architecture. The model was evaluated on an independent test set (*n* = 20) using 3D gamma analysis (3%/3 mm), mean absolute error (MAE), and clinical target volume (CTV) coverage (V_95%_). Mean dose-volume histograms (DVHs) were compared across CTV.

**Results:**

The model achieved a median gamma pass rate of 99.8% within the CTV (range: 78.6–100%), 83.2% outside the CTV (range: 52.3–99.8%), and a whole-volume median pass rate of 90.0% (range: 53.7–99.8%). The median MAE was 0.72 Gy (range: 0.2816–1.8966 Gy). Predicted dose distributions preserved high-dose conformity, with a median of V95% of 97.9% (range: 78.8–100%). DVH curves closely matched the clinical reference plans across all evaluated structures.

**Interpretation:**

The proposed Swin Transformer-based model is a step toward accurate, anatomy-aware dose prediction for brain tumor proton therapy. Future work will address prospective validation and optimization for clinical deployment.

## Introduction

Radiotherapy (RT) planning for brain tumors requires a balance between delivering the prescribed dose to the clinical target volume (CTV) and minimizing exposure to surrounding organs at risk (OARs) [1]. In clinical practice, this is achieved through iterative, manual adjustments by treatment planners, a process that introduces planner‑dependent variability and inter‑institutional inconsistency despite national guidelines [1].

While traditional quality assurance (QA) measures in RT are effective, they do not account for planner-driven deviations, which can lead to inconsistent treatment quality.

Deep learning (DL)-driven dose planning has shown promising results with models demonstrating consistent prediction of realistic, patient-specific, and anatomy-aware dose distributions. DL models for this task have primarily been convolutional neural network (CNN) with generative adversarial networks (GANs), Unets, and variants thereof with strong performance across various anatomical regions [2–4].

The primary focus has been on the development of photon dose prediction, while to a lesser degree in proton therapy. Recently, in pediatric proton therapy of the abdominal region, a 3D UNet achieved average dose differences below 2% [2]. In head-and-neck proton therapy, predicted doses deviated from the clinical plans by –2.53% to –0.12% [5], while [6] reported deviations within 5.1% of the prescription dose. For prostate and lung proton plans, a beam-aware CNN reached voxel-level gamma pass rates of 99.9% in high-dose regions [7, 8].

Lately, vision transformer (ViT) architectures and a variant thereof called a Swin Transformer employ shifted‑window self-attention, which has been proposed for photon dose prediction [9, 10]. Swin Transformer-based models such as Shifted-window UNet Transformer ++ (Swin UNETR++) have reached average DVH score errors as low as 1.6 Gy and achieved 98% patient-wise clinical acceptance on a benchmark photon head-and-neck dataset [10]. In cervical cancer RT, progressive refinement transformer (PRT-Net) demonstrated improved spatial dose conformity in both high-dose target areas and low-dose OAR regions when compared to UNets and DeepLab, which improved long-range dependency modeling and sharper dose structure transitions [9]. CNNs have shown strong performance in proton dose prediction, but their limited receptive field restricts modeling of long-range dependencies, which are an important aspect to proton plans, are highly sensitive to anatomical heterogeneity, and may be more accurately modeled by the properties of Swin Transformers through combining local precision with global context through shifted-window attention [9, 10].

Early studies have begun to explore Swin-based models in photon therapy; however, application of such models targeting proton brain tumor dose prediction remains unaddressed [9, 10].

Therefore, this study investigates whether a Swin Transformer-based DL model can predict voxel-wise dose distributions in proton therapy for brain tumor patients.

## Patients/material and methods

### Data

The patient cohort included 206 individuals diagnosed with primary brain tumors. The group consisted of 51.5% male and 48.5% female patients, and the mean age was 40.6 years. The most frequent tumor types were astrocytoma, oligodendroglioma, and other types of low-grade gliomas. Further details for the patient cohort are presented in Table 1.

**Table 1:** Patient demographic for the training and test data

Parameter	Train/Validation Median (IQR)	Test set Median (IQR)
Number of patients	186	20
Planners/reviewers	11	18
Doses (Gy)	59.4 (50.4–59.4)	59.4 (8.10)
Fractions	33 (28–33)	33.0 (33.0, 29.75)
Number of Beams	3 (3–5)	3.0 (3.0, 3.0)
Age	40.8 (0–81)	37 (24.5) years
Gender	M (51.5%) F (48.5%)	M (53.85.0%) F (46.15%)

IQR: interquartile range.

Proton plans were reviewed and accepted by 11 planners and clinically approved and optimized according to internal dose planning guidelines with constraints defined by the Danish Neuro Oncology Group [11–13]. Compromises between target coverage and normal tissue dose constraints were performed on individual patient basis. A minimum of three fields were used for all plans with a range shifter of 3 to 5 cm for more superficially located tumors. Dose plans were generated in Varian Eclipse (v13.6 and v16.1) with 1 mm dose grid resolution.

For model development, dual-energy computed tomography (CT) scans with corresponding clinical delineations, and the corresponding treatment plans were used. The dataset was randomly split into training (80%), validation (10%), and test set (10%), avoiding selection and stratification bias artificially inflating results.

### Preprocessing

**Figure 1:**
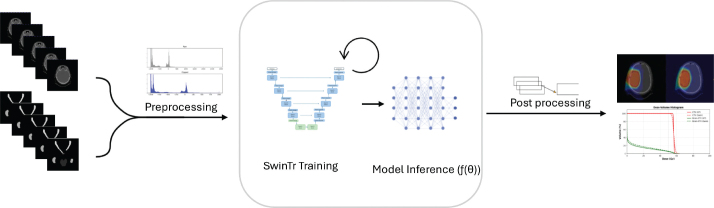
Overview of the proposed dose prediction pipeline using SwinTr. Dual-energy CT images and corresponding structures of the input. The input is followed by preprocessing inputs, which are then used to train a 3D Swin Transformer (SwinTr) embedded in a UNet architecture. After training, model inference is performed to predict the 3D dose distribution. Predicted doses are post-processed through inverse normalization and evaluated using dose–volume histograms (DVHs), gamma analysis, and spatial inspection.

Prior to training, the CT scans and dose plans were preprocessed for standardization. For CT scans, preprocessing involved clipping Hounsfield Unit (HU) values to a predetermined range, followed by intensity normalization to enhance input consistency. Dose plans were normalized to be within a range (0–1) prior to being input to the model. The full processing pipeline is presented in Figure 1.

### Model

3D-shifted-window transformer (SwinTr) within a UNet architecture is proposed as the model . The SwinTr is a hierarchical ViT that uses shifted-window-based self-attention to enable efficient local and global feature extraction with reduced computational complexity. A SwinTr was implemented in a UNet architecture to leverage the strengths of both approaches: the transformer captures complex contextual relationships, while the UNet structure maintains a local receptive field for precise spatial localization [13]. This combination effectively merges global context with spatial detail, as the architecture allows for both hierarchical feature learning and preservation of fine-grained features through skip connections.

The model comprises multiple stages, each containing two transformer blocks, followed by a patch merging operation and L2 regularization. The number of kernels was doubled at each stage of the encoder to progressively increase representational capacity.

**Figure 2:**
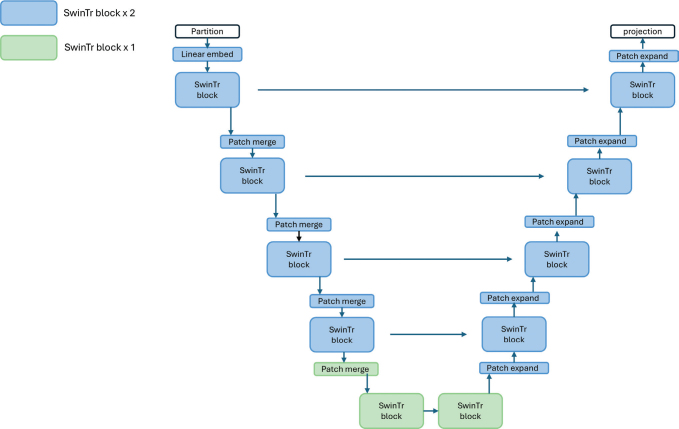
SwinTr-based UNet architecture for 3D dose prediction. The model consists of a symmetric encoder–decoder structure using Swin Transformer (SwinTr) blocks. Each encoder stage includes one or two SwinTr blocks followed by patch merging to reduce spatial resolution and increase feature dimensionality. The decoder mirrors this structure with patch expansion operations to restore resolution.

The decoder mirrored the encoder, using deconvolution layers for up sampling and skip connections to incorporate corresponding encoder features, thereby preserving spatial resolution and structural accuracy in the predicted dose distribution. The architecture is exemplified in Figure 2.

The model is trained in a supervised training protocol and employs the AdamW optimizer with an initial learning rate of 10^-3^, batch size of two, with the loss typically plateauing after 100 epochs. Gradient checkpointing is used to limit memory consumption and enable single Graphic Processing Unit (GPU) training on an NVIDIA A40. Code can be found here.

### Postprocessing

Postprocessing consisted of inverse normalization of the predicted dose plans. During training, all inputs were normalized to a range of (0, 1) for consistency; after prediction, the outputs were rescaled to match the prescribed dose level.

### Evaluation

To evaluate the performance, we employed three-dimensional (3D) gamma analysis, a widely used metric in RT for comparing dose distributions, 3%/3 mm acceptance criteria, where the dose difference must lie within 3% of global maximum dose or the same dose must be found within a distance to agreement of 3 mm. This was performed for the full dose distribution, dose within the CTV and dose outside the CTV [14].

Additionally, the mean absolute error (MAE) was computed for dosimetric comparison. A visual inspection of CTV coverage was also performed using dose-volume histograms (DVHs).

## Results

**Figure 3:**
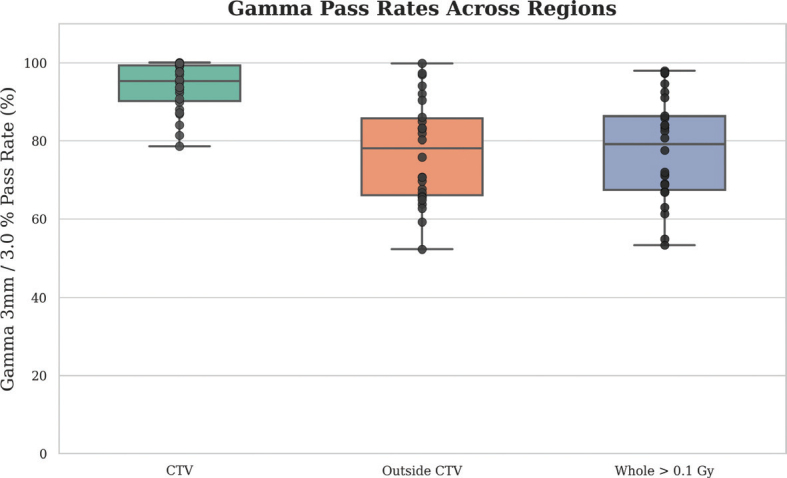
Gamma pass rates with three spatial regions: within the Clinical Target Volume (CTV), outside the CTV, and the entire volume receiving >0.1 Gy. The boxplots show the distribution of pass rates across test patients, with median values exceeding 80% in all regions.

Gamma pass rates for the test set were evaluated across three spatial regions: within the CTV, outside the CTV, and the entire patient volume receiving a dose higher than 0.1 Gy. The highest gamma pass rates were observed within the CTV, with a median of 99.80% (range: 78.6–100.0%). Outside the CTV, gamma pass rates reached a median of 83.2% (range: 52.3–99.8%), while whole-dose gamma pass rates across the entire patient volume achieved a median of 90.0% (range: 53.7–99.8%), as shown in Figure 3.

**Figure 4:**
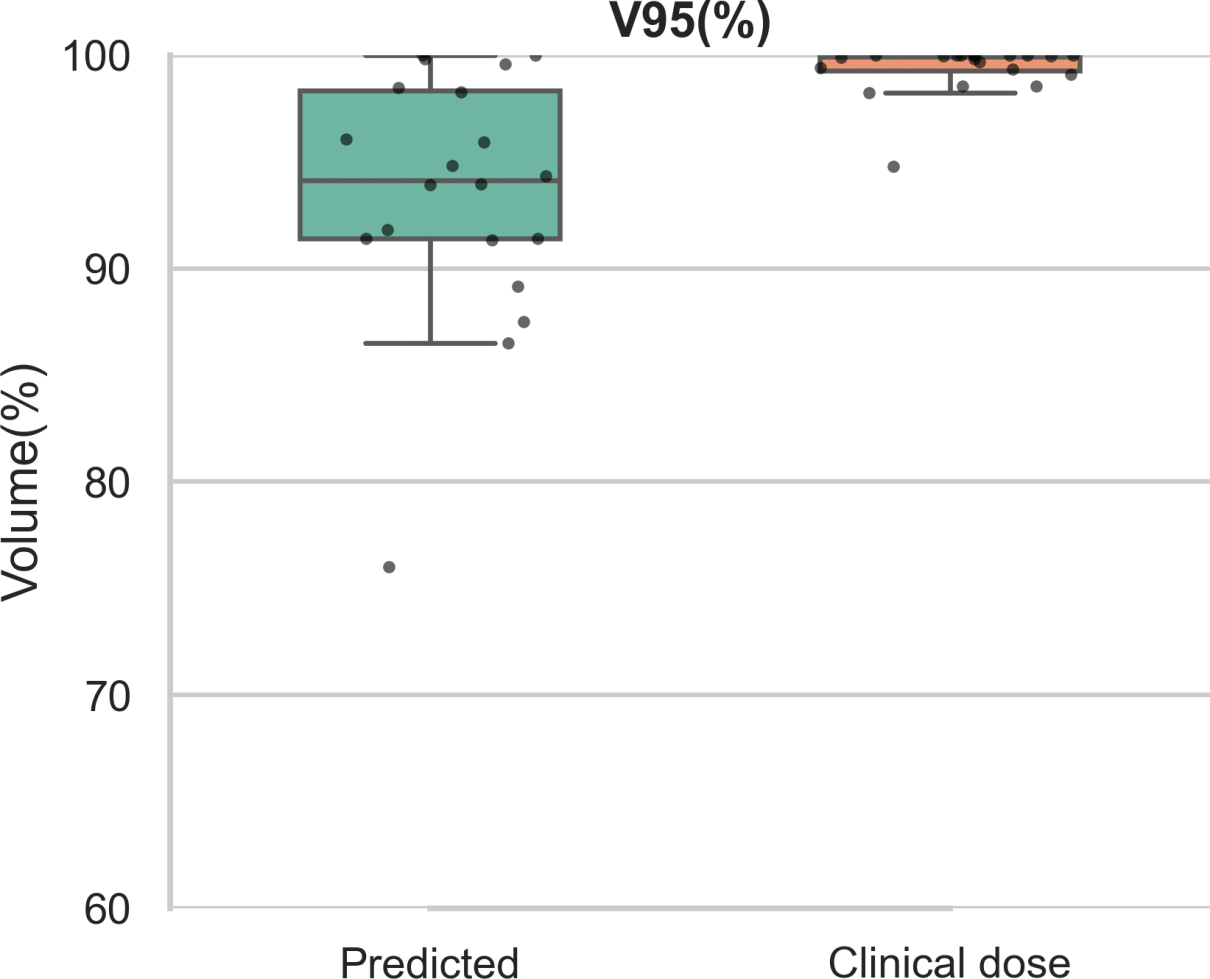
V95 over the Clinical Target Volume (CTV). The boxplot compares the spatial overlap between predicted and clinical dose distributions at the high-dose threshold.

For dosimetric comparison, the global median MAE across all test cases was 0.72 Gy (range: 0.2816 – 1.8966 Gy). To further assess dose conformity within the high-dose region, we evaluated V95% for the CTV which is presented in Figure 4.

A summary of per-patient dose metrics is presented in Table 2. Comparison of mean dose values between the clinical and predicted dose plans revealed relative differences that varied across organs. The Brainstem showed a +36.1% increase, while the Chiasm and Hippocampus R exhibited reductions of −65.6 and −64.8%, respectively. The Pituitary and Hippocampus L were higher in the predicted distributions by +51.3 and +53.9%, respectively. For the optic structures, the Optic Nerve L and R differed by +44.3 and +36.7%, respectively, and the Optic Tract L and R by +30.7 and +28.6%, respectively. Results for these comparisons are summarized in Table 3.

**Table 2:** Clinical and predicted DVH metrics and gamma pass rates for individual patients.

Patient	Clinical DVH					Predicted DVH					Gamma pass rate Within	Gamma pass rate Outside
	Dmean [Gy]	Dmax [Gy]	D98 [Gy]	D95 [Gy]	V95 (%)	Dmean [Gy]	Dmax	D98	D95	V95 (%)		
1	59.39	62.4	58.2	58.51	99.42	59.40	70.19	53.58	55.19	96.03	84.05	67.71
2	59.40	62.45	57.62	58.10	99.33	59.40	69.22	52.67	54.12	99.94	92.28	69.74
3	59.39	62.21	58.43	58.62	100	59.39	67.98	56.11	56.65	99.27	100	94.05
4	59.4	62.342	58.07	58.31	99.99	59.4	68.26	55.26	56.25	97.74	99.92	96.93
5	54	56.45	52.937	53.16	100.0	53.99	69.22	44.18	46.05	78.76	99.83	86.04
6	59.4	62.35	56.34	57.2	99.87	59.399	66.75	54.29	55.29	97.899	93.71	83.22
7	58.1	61.39	57.149	57.317	99.95	59.399	70.57	51.64	54.471	98.29	93.62	69.62
8	59.37	61.99	58.41	58.61	99.95	59.35	66.14	56.156	56.84	99.09	97.69	99.82
9	59.4	62.35	56.34	57.2	99.87	59.4	66.75	54.299	55.296	97.899	93.71	83.22
10	59.399	61.74	57.899	58.136	100	59.4	68.90	55.06	55.966	97.626	99.80	97.34
11	50.4	53.121	49.3	49.31	99,98	59.39	66.62	55.09	56.41	100.00	78.59	53.69
12	59.33	62.66	57.58	58.0	99.1	50.4	63.05	43.346	44.798	87.56	99.80	92.08
13	59.31	63.90	57.97	58.17	99.68	59.4	64.8	55.64	56.19	99.92	81.38	70.76
14	59.32	62.46	58.43	58.60	99.99	59.39	67.94	57.01	57.4295	99.70	99.74	90.42
15	59.33	62.67	57.58	58.01	99.10	59.40	66.86	54.51	55.97	98.83	94.98	75.84
16	59.4	61.91	56.736	58.246	98.24	59.4	68.31	49.74	52.23	100	89.96	52.26
17	50.401	53.144	49.241	49.466	99.84	50.39	60.5	41.81	44.21	89.16	93.08	59.23
18	50.399	53.7	49.105	49.36	99.9	50.399	59.98	44.46	46.07	91.33	97.79	65.77
19	53.857	56.1	52.467	52.776	100	54.0	65.71	47.82	49.33	91.81	99.18	62.75
20	59.398	64.742	54.137	56.321	94.83	59.4	71.1	49.7	52.54	84.20	86.915	83.0

DVH: dose-volume histograms.

Reported metrics include mean dose (Dmean), maximum dose (Dmax), near-minimum dose (D98), near-prescription dose (D95), and target coverage (V95). Gamma analysis was performed with 3%/3 mm criteria, reported separately for within-CTV and outside-CTV regions.

**Table 3:** Comparison of predicted and clinical mean doses across organs-at-risk (OARs).

Organ	Clinical	Predicted mean [Gy]	Difference [%]
Brainstem	8.2	11.16	36.1
Chiasm	16.88	5.81	65.6
Pituitary	10.25	15.51	51.3
Hippocampus L	15.35	23.62	53.9
Hippocampus R	14.2	5.0	64.8
Optic Nerve L	8.05	11.62	44.3
Optic Nerve R	11.42	15.62	36.7
Optic Tract L	15.8	20.64	30.7
Optic Tract R	17.9	23.02	28.6

The table shows mean dose values (Gy) for clinical and predicted plans along with their relative percentage differences.

**Figure 5:**
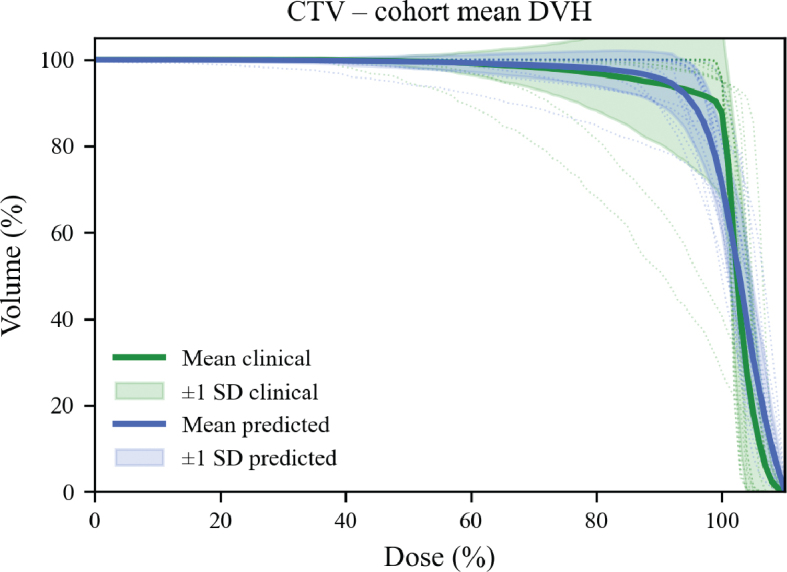
Dose-volume histograms (DVHs) comparing clinical dose and Swin Transformer-based predicted dose distributions across target structures. DVHs are shown for the clinical target volume (CTV), solid lines represent the mean DVH, while dashed lines show the individual DVHs for each patient. Blue lines represent the predicted doses, and green are the clinical dose plans.

**Figure 6:**
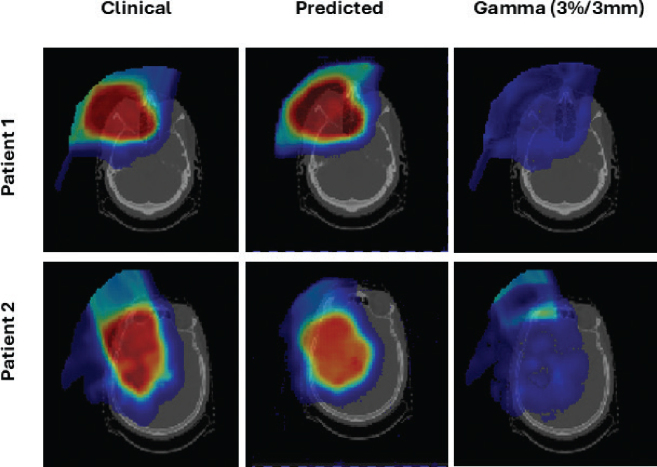
Comparison of clinical dose distribution, predicted dose distribution, and corresponding gamma maps (3%/3 mm criteria) for two representative patients. Rows correspond to different patients; columns correspond to clinical reference dose, model prediction, and gamma evaluation.

DVHs were generated to compare dose distributions across CTV. In Figure 5, solid lines represent ground truth values, and dashed lines indicate predictions. Predicted dose distributions resembled the reference across structures. The individual test patient results are presented in Table 1, and examples of the predicted and clinical dose plans are presented in Figure 6.

## Discussion and conclusion

This study demonstrates that SwinTr-based architectures can accurately predict dose distributions in proton therapy for brain cancer patients. The proposed model achieved a median MAE of 0.72 Gy (range: 0.2816 – 1.8966 Gy), reflecting high similarity to the clinical plans. The gamma pass rate within the CTV reached a median of 99.8% (range: 78.6–100%), and V95% values reached a median of 97.9% (range: 78.8–100%), further supporting the model’s ability to predict plans with high-dose target coverage and minimal deviation.

Evaluation of DVH metrics confirmed these findings while also revealing systematic patterns in model behavior. Across patients, predicted Dmean values remained closely tied to prescription levels (~ 59.4 Gy), often matching the clinical average even when individual plans deviated. At the same time, Dmax tended to be overestimated relative to clinical distributions (e.g. 73.3 Gy vs. 62.2 Gy), whereas coverage-related endpoints (D95, D98) were slightly underestimated in several cases. Most V95% values remained consistent with clinical targets (>95%), though select outlier cases (e.g. Patients 3 and 13) showed reduced coverage, with predicted V95% dropping to 82.3% and 87.6%, respectively.

Differences in mean dose values were generally modest across most organs, whereas larger deviations were observed for the Chiasm and Hippocampus R. This variation could be influenced by the diversity of planning styles represented in the dataset, which included contributions from 11 different planners in the training cohort and 18 in the test cohort. Each planner introduces distinct trade-offs between target coverage and OAR sparing, leading to heterogeneous dose distributions that may be difficult for the model to learn consistently. As a result, discrepancies in OAR endpoints may not solely reflect model limitations and also the intrinsic variability of human planning practice.

Taken together, these patterns suggest that while the SwinTr model reliably reproduces global target coverage and average dose levels, localized discrepancies at high- and low-dose extremes require further attention. In clinical terms, overestimation of Dmax and underestimation of D95/D98 may affect evaluation of hot spots and coverage robustness, particularly in heterogeneous brain anatomy. Mitigating these deviations through loss function refinement and explicit DVH-constrained optimization, could improve clinical reliability and reduce the likelihood of coverage compromise in outlier cases.

Compared to previously reported CNN models, the SwinTr architecture achieves similar performance. Specifically, prior studies have reported MAEs ranging from 1.1 Gy to 4.7 Gy and gamma pass rates between 89.7 and 99.9% [2, 6, 8, 15–18]. The proposed model achieved a lower MAE and similar gamma pass rates with gamma pass rates approaching >90% acceptance, particularly within the CTV across planners, which can potentially be attributed to the global receptive field introduced by shifted-window attention, which enables modeling of long-range dependencies, a critical aspect in proton therapy for brain cancers due to its anatomical heterogeneity and beam sensitivity. We did however see lower gamma pass rates outside the CTV and higher mean dosages to the OARs, which could be attributed to a broad range of planners allowing for inter-planner variation with the test-set including planners not included in the training set. We did test durability and generalizability on out of domain and during inference where plans were still available, suggesting a potentially variable approach for this type of model to generalize across planners and maybe even departments if model and data size increased.

The results align with recent findings for dose prediction of proton plans using transformer-based architectures [10, 15]. Wang et al. [10] employed a SwinTr variant for head-and-neck photon dose prediction and achieved clinical acceptability rates between 90% and 98%. Our model reaches similar accuracy but extends these insights into the less-studied domain of brain proton therapy, representing a step forward for transformer-driven dose.

The results were obtained without stratifying by patient age, gender, and with 10 new planners included in the test cohort. These factors introduce substantial heterogeneity compared to prior benchmark studies, yet the model still achieved sub Gy accuracy and preserved high-dose target coverage. This suggests that the approach is robust under real-world variability and may be more representative of clinical deployment conditions than highly curated datasets.

This study presents limitations that must be acknowledged to strengthen the clinical applicability of the proposed model. The current evaluation was conducted retrospectively, without integration into clinical workflows or validation across multiple institutions. As a result, the model’s performance under real-time planning conditions remains untested. Prospective studies will be essential to assess feasibility, generalizability, and clinical utility. However, from a translational perspective, such robustness is critical for clinical adoption, as proton therapy planning often involves heterogeneous imaging quality, planner experience, and institutional conventions. Once prospectively validated, the model could be integrated as a decision-support tool within the planning workflow either to provide a rapid dose estimation immediately after contouring or to flag plans that deviate from patient-specific dose expectations prior to approval. This could reduce plan review time, improve inter-planner consistency, and support adaptive re-planning in cases of anatomical change. In the longer term, the framework may also aid in patient stratification by identifying those likely to benefit most from proton therapy.

Another consideration is the computational cost associated with Swin Transformer architectures, particularly during training. High memory usage and prolonged training time may pose challenges in resource-constrained environments, which are likely to increase as datasets grow in size and complexity. Future research should explore strategies to reduce computational demands, such as knowledge distillation, model pruning, or transformer-specific optimizations without compromising model accuracy, thereby supporting broader clinical adoption.

## Conclusion

This study demonstrates the potential of a SwinTr-based approach for predicting patient-specific dose distributions in proton RT for brain cancer. The model was evaluated using gamma analysis, MAE, and V_95%_, which showed high anatomical fidelity and low dose difference across the test set.

These findings support the feasibility of using Swin Transformer-based architectures as standardized reference models in proton therapy, potentially useful in guiding dose planner or serving as additional QA. Future work will focus on reducing computational costs for clinical deployment and conducting prospective studies to validate performance under real-world planning conditions.

## Data Availability

Patient-specific data cannot be shared due to the Danish legislation. Technical data can be shared upon reasonable request.
